# Trends in sexual violence patterns and case management: a sex disaggregated analysis in Goma, Democratic Republic of Congo

**DOI:** 10.1186/s13031-021-00398-x

**Published:** 2021-07-23

**Authors:** Justin Paluku Lussy, Annie Dube, Jonathan Kasereka M. Lusi, Aurélien Mahamba Kikoli, Eugénie Kamabu Mukekulu, Susan A. Bartels

**Affiliations:** 1 HEAL Africa Hospital, Goma, North Kivu Democratic Republic of Congo; 2 Department of Obstetrics and Gynecology, UNIGOM: Université de Goma, Goma, North Kivu Democratic Republic of Congo; 3 Northern Ontario School of Medicine, Sudbury, Ontario Canada; 4 Departments of Emergency Medicine and Public Health Sciences, Queen’s University, Kingston, Ontario Canada

**Keywords:** Democratic Republic of Congo, Sexual violence, Post-assault care, Female survivors, Male survivors

## Abstract

**Background:**

Both conflict and non-conflict sexual violence have been well described in the Democratic Republic of Congo (DRC). However, there is little empiric data comparing sexual violence patterns for males and females in the DRC, and little is known about how post-sexual assault care experiences may differ between the two sexes.

**Methods:**

This was a retrospective, registry-based study at HEAL Africa Hospital. Researchers extracted and analyzed available data for all patients seeking post-sexual assault care between July 2013 and December 2017. Comparative analysis was conducted using SAS to document patterns of sexual violence among male and female survivors and to describe the clinical management of males and females seeking post-assault care.

**Results:**

Between July 2013 and December 2017, the hospital provided post-sexual assault care to 1766 patients (1623 female and 93 male). Female survivors were more likely to be minors under the age 18 (*p* < 0.0001) with a mean age 16.5 years versus 22.3 years for males. For both sexes, approximately half of all perpetrators were civilians who were known to the survivor (friends, family members, colleagues or neighbors). After sexual assault, males (79.6%) were more likely than females (55.7%) to present to the hospital within 72 h (*p*-value < 0.0001). Among female patients, 12% had a positive pregnancy test at the time of presentation and another 43% received emergency contraception. Male survivors were more likely to test positive for HIV (*p*-value = 0.0032) and to receive HIV post-exposure prophylaxis as well as prophylactic antibiotics (*p*-value < 0.0001).

**Conclusions:**

In this single-centre registry, non-conflict-related sexual violence affected both women and girls as well as men and boys in North Kivu with civilian-perpetrated assaults being most common, and girls under the age of 18 being disproportionately affected. Overall, delays to seeking post-assault care appear to have decreased over time, although females presented later than males. These differences, as well as sex discrepancies in receiving HIV prophylaxis and prophylactic antibiotics, are not well understood. Additional research is needed to understand these phenomena such that equitable and optimal care can be ensured for both female and male sexual violence survivors.

## Introduction

The Democratic Republic of Congo (DRC) has experienced armed conflict for decades involving the national military as well as foreign and local militia groups. In 2007 it was estimated that war in DRC had been responsible for 5.4 million deaths including largely preventable and treatable conditions such as malnutrition and infections [1]. As a result of these findings, conflict in the DRC was recognized as the deadliest war since World War II [1]. Despite peace agreements and an official end to the war in 2002, armed militia groups continue to perpetrate violence in the eastern regions where there is weak governance and lack of law enforcement [2]. Due to the recent Ebola and COVID-19 outbreaks, as well as spikes in insecurity, 5 million people are reportedly currently displaced within DRC and 15.6 million people (nearly 16% of the population) are in need of humanitarian assistance [3].

Sexual violence has been a defining aspect of the conflict in DRC and a number of studies have brought attention to its use as a weapon of war [2, 4–7]. In a population-based study, Johnson et al. reported that approximately 40% of women in eastern DRC had experienced sexual violence in their lifetime [8] and nationally representative household data from 2007 found that up to 1.8 million Congolese women of childbearing age had experienced sexual violence in their lifetimes [9]. Sexual violence in this context has been associated with adverse physical outcomes including unplanned pregnancies, sexually transmitted infections, as well as pelvic and other traumatic injuries [8, 10]. Additionally, sexual violence survivors in DRC experience high rates of stigma in their communities [11–13] and often face negative socioeconomic outcomes as a result of the sexual assault [14, 15].

Sexual violence against men and boys has been increasingly recognized [16], with recent studies shedding light on its prevalence and consequences in both civilian [17, 18] and conflict settings [18–23]. A 2012 literature review identified that studies on sexual violence against men/boys were more common in armed conflict than in non-conflict settings [18], and Sivakumaran has researched soldier- and armed combatant-perpetrated rape. His work reveals that sexual violence against men/boys is used as a demonstration of power and dominance, and that through rape, victims are emasculated through “feminization” - i.e. that they are perceived to have “lost their manhood” [24]. While all sexual violence is believed to be widely under-reported, this is likely exacerbated for sexual violence against men/boys, which has been touted a ‘human rights violation that is too taboo to talk about’ [25]. Limited availability of services for male sexual violence survivors has also been reported and experiences of stigma have been documented stemming from service providers who have not been sensitized to sex male assault [26, 27].

Current literature on sexual violence in DRC focuses largely on militarized rape [4, 5, 28]. However, it has been recognized to an increasing extent that civilian-perpetrated sexual violence is also a significant problem in DRC [29], with more recent studies signalling increased numbers of sexual assaults perpetrated by non-military personnel [30, 31]. Inequitable gender norms [30, 32], an environment of impunity [4], and a “normalization” of rape in society [5, 29, 30, 32] have likely contributed to this increase in civilian-perpetrated sexual violence. Additionally, with an increased appreciation that men and boys also experience sexual violence, particularly in conflict-affected settings like DRC, further research examining men’s experiences and their access to care and services is urgently needed. Finally, the case management of sexual assaults and how this has evolved over time in eastern DRC has not been well described. Therefore, to address the lack of recent sex-disaggregated data around sexual violence patterns and sexual assault case management in eastern DRC, the current analysis was conducted with the following three objectives: 1) to document patterns of sexual violence among survivors seeking care with a focus on how patterns have changed over time; 2) to describe the current clinical management of sexual assault at North Kivu’s largest sexual violence care centre (HEAL Africa Hospital); and 3) to identify differences in sexual assault patterns and case management between male and female survivors.

## Methods

### Setting

HEAL Africa is a non-governmental hospital in Goma, the capital city of North-Kivu province in eastern DRC. The hospital was established in 2000 to provide medical care and support to those suffering from the devastating consequences of armed conflict. It is a full service hospital offering general surgery, orthopaedics, obstetrics and gynaecology (including fistula repair), paediatrics, internal medicine, radiology and pathology services [33]. It also has community-based initiatives in public health, community development, and conflict resolution, and serves as a research and training centre for healthcare professionals. HEAL Africa offers holistic care to survivors of sexual and gender based violence, and has been recognized as a leading organization for the support of women and girls in the North-Kivu province [34]. HEAL Africa’s comprehensive Sexual Violence Program provides free medical and surgical care to survivors of violence and supports psychosocial and emotional wellbeing, as well as, long term rehabilitation, skills building and community development initiatives.

### Study design and data extraction

A retrospective, registry-based study was conducted at HEAL Africa Hospital in Goma, North-Kivu Province, DRC. All patients seeking care at HEAL Africa’s Sexual Violence Program were routinely asked about the nature of their assaults by a trained Sexual Violence Program staff member. Sexual violence was defined broadly and any patient who self-identified as having experienced sexual violence was offered care. The administered questionnaire collected demographic information such as age, sex, marital status, and home address, as well as data about the sexual assault including sex of the perpetrator, patient relationship to perpetrator, whether the perpetrator was a minor or an adult, whether the perpetrator was a civilian or military personnel, the location of the assault, and the date of the assault. The patient’s medical record documented if there was a chief complaint at time of presentation to the hospital, physical exam findings (i.e. presence of lesions, bruising, redness, semen) and results of relevant tests (pregnancy and HIV). Finally, prophylactic treatments offered to the patient were outlined including anti-retroviral medications to prevent HIV, antibiotics to prevent sexually transmitted infections (STI) and emergency contraception to prevent pregnancy. All data were recorded in French on paper forms, and later entered into a Microsoft Excel spreadsheet by program staff.

### Analysis

In June and July 2018, researchers de-identified and reviewed data from all patients presenting to HEAL Africa’s Sexual Violence Program between July 2013 and December 2017 (representing all data available at that time). Data were translated to English, grouped according to self-identified survivor sex. Comparative analyses were performed using SAS Studio (*Release:* 3.8 Basic Edition, *Site name: University Edition 2.8 9.4 M6*). Chi squared tests were used except where there were cell counts of 5 or less, in which case, Fisher’s exact tests were performed. If data had more than two categories, a left-sided Fisher’s exact test was performed, otherwise a two-sided Fisher’s exact test was used. Stated *p*-values are for chi-square tests unless otherwise noted.

### Ethical considerations

This study was conceptualized by staff of the HEAL Africa Sexual Violence Program and the study protocol was approved by the ethics review board at HEAL Africa. All patient names and identifiers were removed from the database to maintain confidentiality.

## Results

### Demographics of survivors

In total, 1766 patients (1623 female and 93 male) sought post-sexual assault care at HEAL Africa Hospital’s Sexual Violence Program between July 2013 and December 2017. An additional 50 records (2.8%) were missing sex data. The majority of patients presented for care after a single sexual assault, but a small number of patients had experienced multiple assaults. Female survivors were more likely to be minors under the age 18 (*p* < 0.0001) with a mean age 16.5 years versus 22.3 years for males. For both sexes, the majority of survivors were single (73.4% female, 58.1% males), however, males were more likely to be married (25.8%) in comparison to female survivors (6.9%) (*p*-value < 0.0001).

The majority of all survivors (91.6%) were from Goma City with the remainder being from surrounding territories. Table 1 provides full demographics of all patients included in the analysis.

**Table 1:** Demographics and assault characteristics from patients seeking care at HEAL Africa’s Sexual Violence Program between July 2013 and December 2017

	Sexual Violence Survivors								
	No. Total (% of *N* = 1766)		No. Females (% of *N* = 1623)		No. Males (% of *N* = 93)		No. Sex not Identified (% of *N* = 50)		**P-Value**
**Sex of Survivor**									
Female	1623	(91.9)	1623	(100.0)	–	–	–	–	*N/A*
Male	93	(5.3)	–	–	93	(100.0)	–	–	
*Missing data*	50	(2.8)					50	(100.0)	
Total	1766	(100.0)	1623	(100.0)	93	(100.0)	50	(100.0)	
**Age of Survivor** ^**A**^									
Minor	1267	(71.8)	1202	(74.1)	29	(31.2)	36	(72.0)	< 0.0001
Adult	487	(27.5)	412	(25.4)	63	(67.7)	12	(24.0)	
*Missing data* ^*B*^	12	(0.7)	9	(0.6)	1	(1.1)	2	(4.0)	
Total	1766	(100.0)	1623	(100.0)	93	(100.0)	50	(100.0)	
**Marital Status of Survivor**									
Single	1245	(70.5)	1150	(70.9)	54	(58.1)	41	(82.0)	< 0.0001 ^C^
Married	141	(8.0)	112	(6.9)	24	(25.8)	5	(10.0)	
Widowed	27	(1.5)	26	(1.6)	–	–	1	(2.0)	
Divorced	23	(1.3)	23	(1.4)	–	–	–	–	
*Missing data*	330	(18.7)	312	(19.2)	15	(16.1)	3	(6.0)	
Total	1766	(100.0)	1623	(100.0)	93	(100.0)	50	(100.0)	
**Sex of Perpetrator**									
Male	1300	(73.6)	1263	(77.8)	34	(36.6)	3	(6.0)	< 0.0001
Female	89	(5.0)	12	(0.7)	33	(35.5)	44	(88.0)	
*Missing data*	377	(21.3)	348	(21.4)	26	(28.0)	3	(6.0)	
Total	1766	(100.0)	1623	(100.0)	93	(100.0)	50	(100.0)	
**Age of Perpetrator** ^**A**^									
Adult	1200	(68.0)	1098	(67.7)	64	(68.8)	38	(76.0)	0.1063 ^D^
Minor	151	(8.6)	142	(8.7)	3	(3.2)	6	(12.0)	
*Missing data*	415	(23.5)	383	(23.6)	26	(28.0)	6	(12.0)	
Total	1766	(100.0)	1623	(100.0)	93	(100.0)	50	(100.0)	
**Status of Perpetrator**									
Civilian	928	(52.5)	868	(53.5)	46	(49.5)	14	(28.0)	0.1667
Military	244	(13.8)	232	(14.3)	7	(7.5)	5	(10.0)	
*Missing data*	594	(33.6)	523	(32.2)	40	(43.0)	31	(62.0)	
Total	1766	(100.0)	1623	(100.0)	93	(100.0)	50	(100.0)	
**Relationship between Perpetrator and Survivor**									
Friend	307	(17.4)	291	(17.9)	15	(16.1)	1	(2.0)	*Inconclusive due to low cell count
Neighbour	250	(14.2)	242	(14.9)	8	(8.6)	–	–	
Stranger	118	(6.7)	113	(7.0)	5	(5.4)	–	–	
Family member ^E^	68	(3.9)	68	(4.2)	–	–	–	–	
Colleague	29	(1.6)	21	(1.3)	8	(8.6)	–	–	
Authority Figure ^F^	18	(1.0)	18	(1.1)	–	–	–	–	
Bandit	10	(0.6)	10	(0.6)	–	–	–	–	
Driver	2	(0.1)	2	(0.1)	–	–	–	–	
*Missing data*	964	(54.6)	858	(52.9)	57	(61.3)	49	(98.0)	
Total	1766	(100.0)	1623	(100.0)	93	(100.0)	50	(100.0)	

Notes. ^A^ Age is classified as either minor: under 18 years or adult: 18 + years. ^B^ Missing data were not included in the chi-square or Fisher’s exact tests. ^C^ Fisher’s exact left-sided *p*-value given low cell counts. ^D^ Fisher’s exact two-sided *p*-value given low cell counts. ^E^ Family members include cousin, husband, uncle and sibling. ^F^ Authority figures include police officer, employer, director, pastor, professor

### Characteristics of perpetrators

For sexual violence female survivors, 77.8% of perpetrators were male, whereas, for male survivors of sexual violence, 36.6% of perpetrators were male (*p*-value < 0.0001). No statistically significant differences were seen between female and male survivors regarding the age or status (civilian or military) of the perpetrator. The majority of perpetrators were adult (67.7% for female survivors and 68.8% for male survivors) and civilian (53.5% for females and 49.5% for male survivors of sexual violence). For both female and male survivors, the perpetrators were most commonly known to them as a friend (17.9% for female survivors and 16.1% for male survivors) or neighbor (14.9% for female survivors and 8.6% for male survivors). Additional characteristics of the sexual assaults are provided in Table 1.

### Circumstances of the assault

Although some survivors reported the circumstances around their sexual assaults, 1270 data points (71.8%) were missing. For female survivors, of the 444 reported data values, sexual assault most commonly occurred while visiting or relaxing with a friend (19.6%), on the road and perpetrated by bandits (14.9%) or military personnel (4.7%), or during a family event by a neighbor (16.7%). For male survivors however, of the 17 reported data values, sexual assaults occurred on the road and perpetrated by bandits (41.2%), at home and perpetrated by family members (11.8%) or while intoxicated (11.8%). Other assault circumstances experienced by both female and male survivors included while working in the fields or farming, at home and perpetrated by bandits or military personnel, in the workplace and perpetrated by employers, colleagues or clients, while receiving care from healthcare providers, in prison, at church or school, at a store or restaurant, while walking home late at night, while collecting water or firewood, while engaging in commercial sex work or during a kidnapping.

### Care at HEAL Africa hospital

Of those presenting to HEAL Africa for post-sexual violence care, male survivors (*n* = 74, 79.6%) were more likely than female survivors (*n* = 904, 55.7%) to present within 72 h of the assault occurring (*p*-value < 0.0001). The majority of survivors (92.9% for female and 73.1% for male) presented with sexual violence and / or exploitation as their chief complaint. However, male survivors were more likely to present with a chief complaint other than sexual violence (*p*-value < 0.0001). This included kidnapping, exposure to blood and / or other bodily fluids and physical abuse. Additional details are displayed in Table 2.

**Table 2:** Sexual Assault Case Management at HEAL Africa’s Sexual Violence Program between July 2013 and December 2017

	Sexual Violence Survivors								
	No. Total (% of *N* = 1766)		No. Females (% of *N* = 1623)		No. Males (% of *N* = 93)		No. Sex not Identified (% of *N* = 50)		**P-Value**
**Seen in 72 h**									
Yes	1004	(56.9)	904	(55.7)	74	(79.6)	26	(52.0)	< 0.0001
No	760	(43.0)	718	(44.2)	19	(20.4)	23	(46.0)	
*Missing data*	2	(0.1)	1	(0.1)	–	–	1	(2.0)	
Total	1766	(100.0)	1623	(100.0)	93	(100.0)	50	(100.0)	
**Chief Complaint**									
Sexual violence	1622	(91.8)	1507	(92.9)	68	(73.1)	47	(94.0)	< 0.0001
Other	113	(6.4)	91	(5.6)	21	(22.6)	1	(2.0)	
*Missing data*	31	(1.8)	25	(1.5)	4	(4.3)	2	(4.0)	
Total	1766	(100.0)	1623	(100.0)	93	(100.0)	50	(100.0)	
**HIV Testing**									
Negative	1460	(82.7)	1352	(83.3)	67	(72.0)	41	(82.0)	0.0032 ^A^
Positive	41	(2.3)	34	(2.1)	4	(4.3)	3	(6.0)	
Not performed	25	(1.4)	20	(1.2)	5	(5.4)	–	–	
*Missing data*	240	(13.6)	217	(13.4)	17	(18.3)	6	(12.0)	
Total	1766	(100.0)	1623	(100.0)	93	(100.0)	50	(100.0)	
**Anti-Retroviral Medication**									
Yes	982	(55.6)	882	(54.3)	74	(79.6)	26	(52.0)	< 0.0001
No	683	(38.7)	650	(40.0)	13	(14.0)	20	(40.0)	
*Missing data*	101	(5.7)	91	(5.6)	6	(6.5)	4	(8.0)	
Total	1766	(100.0)	1623	(100.0)	93	(100.0)	50	(100.0)	
**Antibiotic Medication**									
Yes	1085	(61.4)	985	(60.7)	74	(79.6)	26	(52.0)	< 0.0001
No	579	(32.8)	547	(33.7)	12	(12.9)	20	(40.0)	
*Missing data*	102	(5.8)	91	(5.6)	7	(7.5)	4	(8.0)	
Total	1766	(100.0)	1623	(100.0)	93	(100.0)	50	(100.0)	

Notes. ^A^ Fisher’s exact left-sided p-value given low cell counts.

For female sexual violence survivors, a pelvic exam was performed to inspect for injuries and to document the presence of semen. Semen was visually identified in 37 female survivors (2.3%) and absent in 1049 female survivors (64.6%). Additional physical exam findings consistent with sexual abuse included recent hymeneal tear (40.7%), pregnancy (13.8%) and vulvar trauma (13.0%). Other findings on physical exam included genital infections (5.5%) and muscular contusions (3.7%). Another 10.1% of female survivors had physical exam findings of sexual abuse with an intact hymen, with a further 2.8% having normal anatomical findings (which did not rule out sexual assault). Finally, 2.8% of female survivors were described as having an unstable psychological state.

Male physical exam findings consistent with sexual assault included the presence of blood and / or other bodily fluids (16.1%), anal lesions, bruising, erythema and fissures (10.8%), generalized bruising, swelling and lesions over the entire body (6.5%) and bruising, lesions and erythema on the penis, scrotum and anus (4.3%). Other findings included fever (2.2%) and unstable psychological status (1.1%). Normal anatomical findings were seen in 42 (45.2%) male survivors, however this did not rule out sexual assault, and 3 (3.2%) male survivors declined the physical exam.

Female survivors were offered urine pregnancy tests and emergency contraception if indicated. Children under the age of 12 as well as survivors who were already known to be pregnant or menopausal at the time of the assault were excluded from pregnancy tests. Emergency contraception was not offered to women who presented > 120 h after the sexual assault. Two hundred female survivors (12.3%) had a positive pregnancy test, 776 (47.8%) had a negative pregnancy test and 315 (19.4%) did not undergo pregnancy testing. Lastly, emergency contraception was provided to 691 (42.6%) female survivors and not provided to 838 (51.6%) female survivors.

Case management for both female and male sexual violence survivors presenting to HEAL Africa included serum HIV testing and prophylaxis. HIV results were classified according to whether the test was performed and, if yes, what the results were (positive or negative). The distribution of HIV results varied between female and male survivors (*p*-value = 0.0032). For female survivors, 1352 HIV tests were negative (83.3%), 34 were positive (2.1%) and 20 were not performed (1.2%). For male survivors, 67 HIV tests were negative (72.0%), 4 were positive (4.3%) and 5 were not performed (5.4%). Similarly, differences between female and male survivors were observed for the administration of HIV post-exposure prophylaxis (PEP) and antibiotics to prevent STI (*p*-value < 0.0001 for each comparison). HIV PEP was administered to 79.6% of male survivors and 54.3% of female survivors. Prophylactic antibiotics were administered for STIs, including to 79.6% of male survivors and 60.7% of female survivors (p-value < 0.0001).

All survivors received counseling and psychological support. Table 2 displays additional results from the clinical investigations and medical management.

Other medical and / or surgical treatment, including for traumatic injuries, was provided on a case by case basis. Those who requested legal assistance were attended to by dedicated program lawyers.

## Discussion

The current analysis examines patterns of sexual violence and care delivery among male and female patients presenting to HEAL Africa Hospital for post-assault care in Goma, the capital city of North Kivu province. Overall, the results highlight that female survivors were younger than male survivors. For both males and females, approximately half of all sexual assaults were perpetrated by civilians who were often known to them as friends, family members, colleagues and neighbours. While men were responsible for most sexual violence perpetrated against females, male survivors reported roughly equal numbers of male and female perpetrators. With regards to care seeking behaviour and care delivery, men were more likely to present for care within the recommended 72-h window following assault and were more likely to receive HIV PEP as well as prophylactic antibiotics. Approximately 43% of female survivors received emergency contraception and about 12% had positive pregnancy tests at the time of the index visit.

Although sexual violence has been widely described as a weapon of war in eastern DRC [35], trends over time have suggested that civilian-perpetrated sexual violence has increased. For instance, a study from Bukavu in South Kivu province concluded that from 2004 to 2008 the number of sexual assaults reportedly perpetrated by civilians increased by 17-fold (*P* < 0.0001) [5]. Additionally, an analysis of sexual violence survivors presenting to HEAL Africa Hospital between 2006 and 2008 revealed that 77% of patients reported being assaulted by a civilian, who was often known to the survivor [30]. These data, in conjunction with the results presented here, add to the growing body of literature that sexual violence is not just a weapon of war in DRC, and call for recognition of non-conflict-related sexual violence as a pervasive public health and human rights issues in the daily lives of Congolese. It is also critical to recognize that sexual violence against adolescent girls is a particular concern and that men and boys are affected in addition to women and girls.

Multiple studies have documented delays in care-seeking following sexual assaults in the DRC. For instance ﻿in South Kivu province, the international non-governmental organization, Malteser, described significant delays of up to a year or longer between sexual assaults and presentation to care for women who accessed services in 2005 [36]. Another study in Bukavu reported a mean of 16 months and a median of 11 months for sexual violence survivors seeking post-assault care at Panzi Hospital [37]. That 57% of survivors in the current study sought medical care within the recommended 72 h time frame represents a significant improvement from those earlier studies, although we do not have the data to make direct comparisons. If more sexual violence survivors are indeed seeking post-assault care within the recommended 72-h window, this may result from awareness raising campaigns at the community level to highlight the importance of seeking care early when emergency contraception, HIV PEP and antibiotic prophylaxis are more effective.

Our analysis is one of the first to compare post-assault care delivery between male and female sexual violence survivors and several findings are noteworthy. First, men were more likely to present for care within the recommended 72-h time window following assault. The reason for this is unclear from the current data. It may be that male victims experienced more traumatic injuries or that they were aware of the increased risks of HIV and STI transmission through receptive anal intercourse, and therefore sought care including HIV and STI prophylaxis. Since existing gender inequities in the DRC mean that men are often the family’s decision-makers even when it comes to women’s health and care seeking [38], it is also possible that women and girls were not able to seek care without disclosing the sexual assault to the male household head, which may have created a delay. Second, men were more likely to have a positive HIV test. This finding might be expected based on the well documented fact that anal intercourse carries the highest risk of transmitting HIV. However, because 80% of male survivors sought care within 72 h of the sexual assault, it is unlikely that they would have seroconverted in such a short time frame. Instead, the male survivors with positive HIV tests were likely already HIV positive at the time they were assaulted. Third, male survivors were more likely to be treated with HIV PEP and prophylactic antibiotics. Given that anal intercourse carries a higher risk of HIV and STI transmission, it is possible that men were more likely to be offered prophylaxis on that basis.

Much of what has been documented about men’s post-sexual assault care has focused on lack of available services that consider the specific needs of male sexual violence survivors in addition to their experiences of being stigmatized by health care providers [21, 26, 39, 40]. In a qualitative study from South Kivu, DRC, Christian et al. identified priority needs for male sexual and gender-based violence survivors and their families. Quality healthcare, addressing more than physical needs, including individual and / or family counseling, family mediation for reintegration, and ongoing follow up for chronic complications of sexual assault were all recommended [21]. Additionally, Christian et al. found that no male survivors were working outside the home, demonstrating that economic opportunities, community awareness and education were crucial to supporting male survivors [21]. Given the retrospective nature of the current study we were not able to document challenges and barriers to accessing post-assault care or experiences of stigma. However, we recommend future study in North Kivu to examine these important issues and note that it would be important for such a study to also reach men and boys who were not able to, or choose not to, access care.

In their study of gender-based violence against men and boys in the Darfur conflict, Ferrales et al. highlighted how sexual violence ‘homosexualized’ and ‘feminized’ male victims, and that in doing so sexual violence was used to emasculate “African” tribes in Darfur [22]. Unfortunately, the current study design does not allow us to comment on homosexualization and/or feminization of male victims, and we recommend follow up qualitative research to better understand to what degree this phenomenon also exists in DRC. Genital harm was also identified as a prominent theme of conflict-related sexual violence in Darfur with reports of injuries to victims’ testicles and penises which the authors interpreted as demonstrating the association between hegemonic masculinity and men’s sexual organs [22]. Similar accounts of genital torture, mutilation and trauma were not identified in the current hospital registry in eastern DRC. This is likely due, at least in part, to the fact that the Darfurian and Congolese contexts were quite different at the time the respective data was collected and also due to the fact that approximately half of the sexual assaults in the current database were perpetrated by civilians (i.e. were not conflict-related).

Another study relevant to the current work was a ﻿nationally representative sample of sexual assaults perpetrated against men and boys in South Africa and reported to the police in 2012. Similar to the current analysis, in the South African study most of the 209 sexual assaults were perpetrated by someone known to the survivor (57.5%) [41]. However, in contrast to the current work where 31.2% of male survivors were minors under the age of 18, in South Africa ﻿57.4% of the assaults were children. From Jina, et al., it is also interesting that adult survivors were more likely to report the assault immediately, in contrast to minors who were more likely to report > 72 h after the assault (41.2% v. 8.6%) [41]. Finally, adult males in the South African study experienced more violent rapes that were more likely to involve the use of physical force. Similar findings were not revealed in the current analysis although it is possible that the findings from the hospital registry were not documented in sufficient detail to detect such trends. We did not identify any prior research that examined rates HIV PEP and prophylactic antibiotics for male sexual assault survivors although a set of useful guidelines for the medical care of male sexual violence survivors was published for the Middle East and North Africa region [42].

The findings of this study must be interpreted within the context of a number of limitations. First, the data are derived from a single-centre hospital registry and therefore are limited by sampling bias. Sexual violence survivors who may have not sought post-assault care at all or those who may have sought care elsewhere may represent a different demographic or may have had different assault patterns or care experiences. Our results are therefore not generalizable beyond the subset of sexual violence survivors who received post-assault care at HEAL Africa Hospital between July 2013 and December 2017. Additionally, given the retrospective nature and the fact that data were derived from a hospital registry, there was a significant amount of missing data on some variables, which may have altered the results in ways that are difficult to predict. The study also has a number of noteworthy strengths. To our knowledge, it is the first sexual violence study from the African context to include both male and female survivors and to conduct an analysis disaggregated by sex. In doing so, the results provide new insights into sexual violence patterns in North Kivu province. Furthermore, the current research highlights some new differences between males and females with respect to care seeking behaviour and delivery of services. These sex differences, which we have not found reported elsewhere, may have implications for future care delivery if they are substantiated in further research.

## Conclusions

Non-conflict-related sexual violence is a pervasive public health and human rights issue in North Kivu, affecting both women and girls as well as men and boys, and with girls under the age of 18 disproportionately affected. The current sex disaggregated analysis revealed that males were more likely than females to present to the hospital within the recommended 72 h post-assault. Furthermore, male survivors were more likely to have positive HIV tests at the time of post-assault care and were more likely to receive HIV PEP as well as prophylactic antibiotics. These findings may have implications for delivery of equitable services and for ensuring that both female and males sexual violence survivors receive timely and evidence-based care. Additional research, including qualitative studies with survivors, is needed to understand care-seeking behaviour in an effort to further reduce delays to delivery of preventative medical interventions. Research with health care providers would be helpful in elucidating why men and boys were more likely to be treated with HIV PEP and antibiotics following sexual assault.

## Data Availability

The dataset used and/or analyzed during the current study are available from the corresponding author on reasonable request.

## Abbreviations

COVID-19: Coronavirus Disease 2019
DRC: Democratic Republic of Congo
HIV: Human Immunodeficiency Virus
PEP: Post-exposure prophylaxis
STI: Sexually transmitted infection
